# Evaluation of the levels of salivary paxillin in oral potentially malignant disorders and malignant lesions

**DOI:** 10.1186/s12903-024-04569-z

**Published:** 2024-07-24

**Authors:** Amal A. Hussine, Khaled Selim, Olfat Shaker, Yasmine Kamal

**Affiliations:** 1 Oral Medicine and Periodontology Department, Faculty of Dentistry, Cairo University, Cairo, Egypt; 2 Faculty of Medicine, Cairo University, Cairo, Egypt

**Keywords:** Saliva, Oral potentially malignant lesions, Oral squamous cell carcinoma, Paxillin

## Abstract

**Background:**

The scientific community has been particularly interested in oral squamous cell carcinoma **(OSCC)** because of the cancer’s extremely high incidence and fatality rates worldwide. It has been proposed that paxillin is involved in certain malignancies as an oncogene. Additionally, several investigations have assessed paxillin expression and investigated its function in developing distinct human carcinomas, including squamous cell carcinoma. Furthermore, it was discovered that there is a strong link between paxillin levels and cancer progression and spread.

**Objective:**

This investigation was carried out to analyze and compare the salivary paxillin levels between oral potentially malignant disorders **(OPMDs)**, OSCC and the healthy controls to assess its potential role as a biomarker of oral cancer aiming for early diagnosis and better prognosis of OSCC.

**Methods:**

Forty-five patients, ranging in age from thirty to seventy-five, were divided into three groups: fifteen patients with OPMDs, fifteen patients with OSCC, and fifteen controls. Paxillin was identified in saliva samples by using an ELISA kit.

**Results:**

Patients with OSCC and OPMDs have considerably greater salivary Paxillin levels than the healthy control group. The receiver operating curve (ROC) analysis was used in our study to distinguish patients with OPMDs from those with OSCC. The ROC curve constructed with the OPMDs group as the positives had lower sensitivity and area under the curve **(AUC)** values [100% and 1] than the ROC curve with the malignant group as the positives [93.3% and 0.997], respectively. Furthermore, ROC analysis performed between OPMDs group and the malignant group showed a specificity of 73.3% and a cut-off value **≥** 7.9 .

**Conclusion:**

Paxillin can be considered a reliable biomarker for identifying and comparing OPMDs and OSCC cancerous changes.

**ClinicalTrials.gov Identifier:**

NCT06154551- 4/12/2023.

## Background

The tenth most prevalent cancer in the world is OSCC. The diagnosis of OSCC is still challenging, particularly for malignancies that are at stages of progression. One of the main causes of cancer fatal incidents is the intricate process of OSCC metastasis. Whereas tests for early diagnosis, prognosis prediction, and novel therapy approaches for OSCC are easily obtainable, the 5-year survival rate is still poor, which mandates the identification of valuable biomarkers [1]. Delays in diagnosing OSCC have severe ramifications, including detrimental effects on prognosis, patient’s quality of life, and the financial strain on healthcare systems worldwide [2].

The two-step progression of oral cavity cancer has been well documented, and as a result, the majority of malignant ulcers are preceded by early lesions [3]. A potentially malignant lesion is a condition or finding that could develop into malignancies if not appropriately handled. Controlling OPMDs, which affect approximately 2.5% of the general population, is essential to controlling the spread of malignancies. Since the malignant transformation and progression rate of OPMDs can reach 17% within a mean of 7 years after diagnosis. Identifying them as soon as possible is critical, especially for more vulnerable individuals [4].

The most common precancerous disorder of the oral cavity is oral leukoplakia (OL) [5, 6], while one of the most notable indicators of potential future malignancies is epithelial dysplasia [7]. Compared to non-dysplastic lesions, dysplastic OL has a five-fold increased risk of developing cancer [5, 8]. Pathologic examination reveals dysplasia in up to 30% of leukoplakic lesions, but only a small percentage of lesions have invasive SCC. It is significant to highlight that white and keratotic lesions are found in 60% of oral mucosa carcinomas [7].

Lichen planus, a rather common oral mucosal lesion, is controversial regarding its premalignant potential [9]. Differentiating between epithelial dysplasia and lichen planus is typically difficult. According to one study, 6% of oral lichen planus patients met none of the 12 WHO diagnostic criteria for epithelial dysplasia, while 24% met all of them. A few studies that included people with lichen planus who were followed up over time found a higher risk of malignant transformation. Therefore, it is necessary to investigate the lesion during the first visit to rule out any concerns of malignancy [10]. However, the disease is so widespread that it is not feasible to monitor every patient identified with the condition [4].

Therefore, saliva has been proposed as a potential stand-in for non-invasive diagnostic techniques for the early diagnosis, monitoring, and detection of OPMDs and OSCCs. That is, using the establishment of important biomarkers [11].

Paxillin is a connector or a scaffolding protein that aids in bringing together several cytoskeleton and messenger proteins into a complex. In addition to that, it guides the transmission of subsequent signals. It is a protein with numerous domains that localizes primarily to the extracellular matrix’s focal adhesion sites, which are the regions for cell adhesion. Actin filaments are directly bonded to integrin-rich cell attachment sites by paxillin. The primary role of paxillin is to act as a bridging protein that assists in carrying out changes in the organization of the actin cytoskeleton. This function tends to be crucial for the cell locomotion involved in tumor dissemination [12].

Paxillin dynamically operates cytoskeletal reconfiguration and integrates various signals from the extracellular matrix **(ECM)**. That is to maintain cell structure, spreading, motility, proliferation, apoptosis, and vascular development [12, 13]. Paxillin can disrupt normal cell adhesion regulation in tumor cells by acting as a loading site and substrate for several growth factors and oncogenic proteins such as v-src, v-crk, and BCR/ABL [14]. The extracellular matrix must be broken down for tumor cells to penetrate and spread throughout the lymphatic and circulatory systems [15].

A poor prognosis and advanced-stage lymph node metastases are linked to elevated expression of paxillin [12]. It encourages lung cancer and laryngeal cancer to proliferate and spread [16], Kaposi’s carcinoma, [17] and salivary adenoid cystic carcinoma [18].

Furthermore, because of apoptosis stopping and a higher cell survival rate, increased production of Paxillin and its phosphorylated form is thought to be one of the causes of cetuximab’s inadequate therapeutic impact [13, 19].

Paxillin expression in OLP and leukoplakia has yet to be studied. As a result, the primary goal of this research was to compare paxillin expression levels in OSCC and OPMDs to those in healthy people.

## Aim of the study

Thus, the primary goal of this study was to compare paxillin expression levels in OPMDs and OSCC to those in healthy subjects.

## Methods

Forty-five participants between 30 and 70 were accepted for this study. They were registered in the Oral Medicine and Periodontology department of Cairo University’s Faculty of Dentistry’s outpatient clinic. Three sets of subjects were assigned: fifteen patients with OPMDs, fifteen patients with OSCC, and fifteen systemically healthy control subjects. A complete medical history was obtained for each individual using a modified Cornell Medical Index questionnaire. Upon receiving clarification regarding the scope of the inquiry, each participant completed an informed consent form. The study protocol was approved by the Research Ethics committee, Faculty of Dentistry, Cairo University ( approval NO. 32 11 22). All clinicopathological information as gender, age, site of lesion, and tumor grading, habits (smoking, alcohol) was obtained from the medical records and presented in (Tables 1 and 2).

Patients in the three groups were clinically diagnosed, and biopsy specimens from the lesions were taken to confirm the diagnosis based on the histological findings as clarified by Warnakulasuriya et al. [20].

**Table 1:** Intergroup comparison of demographic data

Parameter			Malignant	Potentially malignant	Healthy	Test Statistic	*p*-value
Gender	Male	*n*	7	6	7	**0.18**	**1**
		%	46.7%	40.0%	46.7%		
	Female	*n*	8	9	8		
		%	53.3%	60.0%	53.3%		
Age (years)	Mean ± SD		53.27 ± 7.72	49.33 ± 10.99	50.10 ± 7.13	**0.85**	**0.435**
Smoking	No	*n*	10	8	12	**2.40**	**0.301**
		%	46.7%	40.0%	46.7%		
	Yes	*n*	5	7	3		
		%	53.3%	60.0%	53.3%		
Alcohol	No						

**Table 2:** Descriptive data for malignant group

Parameter			Value
Site	Lip	*n*	1
		%	6.7%
	Tongue	*n*	5
		%	33.3%
	Floor of the mouth	*n*	1
		%	6.7%
	Hard palate	*n*	2
		%	13.3%
	Mandible	*n*	2
		%	13.3%
	Maxilla	*n*	4
		%	26.7%
Tumor grade	Grade (I)	*n*	1
		%	6.7%
	Grade (II)	*n*	9
		%	60.0%
	Grade (III)	*n*	5
		%	33.3%
Lymph node metastasis	No	*n*	12
		%	80.0%
	Yes	*n*	3
		%	20.0%
Blood metastasis	No	*n*	14
		%	93.3%
	Yes	*n*	1
		%	6.7%

### Collection of saliva samples

Before any therapy, entire unstimulated saliva was collected from all subjects. All participants were asked to refrain from drinking, eating, or chewing gum for at least 60 min before sampling. Samples were collected by asking the patient to swallow first, then lean the head forward for 5 min and spit the entire saliva into 50-ml sterile centrifuge tubes. After being assembled, all specimens were immediately stored at -20 ºC until tested.

### Quantitation of human paxillin salivary level

Saliva was collected from patients and controls. After centrifugation, the supernatant was separated for detection of Paxillin by using the ELISA technique provided by Cloud Clone Corp, Houston, USA, Catalogue Number: E-01184hu. This kit’s microtiter plate has been pre-coated with a paxillin-specific antibody. The relevant microtiter plate wells are then treated with a biotin-conjugated antibody specific to paxillin. Then, Avidin conjugated to Horseradish Peroxidase **(HRP)** is added and incubated in each microplate well. After adding the TMB substrate solution, only the wells containing paxillin, biotin-conjugated antibody, and enzyme-conjugated Avidin will change color. The enzyme-substrate reaction is stopped by adding a solution of sulphuric acid, and the color change is measured spectrophotometrically at 450 nm 10 nm. The samples’ optical density **(OD)** is then compared to the reference curve to determine their paxillin concentration.

### Statistical analysis

Categorical data were given as frequency and percentage values, and Chi-square test was used to assess them. Mean and standard deviation **(SD)** numbers were used to depict numerical data. The Shapiro-Wilk test was used to determine their normalcy. A one-way ANOVA test followed by Tukey’s post hoc test was used to analyze normally distributed data (age and Paxillin (ng/mL) levels). ROC curve analysis was done to determine diagnostic accuracy. The best cut-off value was determined using Youden index [21]. A z-test was used to compare ROC curves. The significance level was set at *p* < 0.05 for all tests. R statistical analysis software version 4.3.1 for Windows was used for the statistical analysis [22].

## Results

The study was conducted on 45 cases (i.e., 15 cases per group). There were 7(46.7%) males and 8(53.3%) females in the malignant and healthy groups. There were 6 (40.0%) males and 9 (60.0%) females in the potentially malignant group. The malignant group’s average age was (53.27 ± 7.72) years. In the potentially malignant group, it was (49.33 ± 10.99) years, while in the healthy group, it was (50.10 ± 7.13) years. Gender (*p* = 1) and age (*p* = 0.435). Differences between the tested groups were insignificant. Similarly, no significant difference was detected regarding smoking between participants in the three groups (*p* = 0.301) (Table 1).

The results of Paxillin (ng/mL) intergroup comparisons, revealed a significant difference between the tested groups (*p* < 0.001). The malignant group had the highest mean value, followed by the potentially malignant group, while the healthy group had the lowest value. All pairwise comparisons were statistically significant (*p* < 0.001) (Table 3).

Results of ROC curve analysis presented in Table 4 showed that a ROC curve constructed with the malignant group as the positives had higher sensitivity and area under the curve (AUC) values [100% and 1] than a ROC curve constructed with the potentially malignant group as the positives [93.3% and 0.997] respectively. They also showed that both curves had specificity and cut-off values of [100% and ≥ 6.2], respectively, and that no notable difference was observed in both (AUCs) (*p* = 0.480).

Another ROC analysis was performed between OPMDs group and the malignant group where its results clarified the discriminative ability of the marker between the two groups, showing a specificity of 73.3% and a cut-off value ≥ 7.9 Table 5.

**Table 3:** Intergroup comparison of Paxillin (ng/mL) level

Paxillin (ng/mL) (Mean ± SD)			*F* value	*P* value
Malignant	Potentially malignant	Healthy		
8.99 ± 1.69^A^	7.01 ± 0.45^B^	5.60 ± 0.45^C^	**30.09**	< 0.001*

Means in the same horizontal row with different superscript characters are significantly different *significant (*p* < 0.001)

**Table 4:** ROC curve analysis (each group vs. controls)

Parameter		Malignant	Potentially malignant	AUC difference [95%CI]	*Z* value	*P* value*
Sensitivity [95% CI]		**100.0 [78.2–100.0]**	**93.3 [68.1–99.8]**	**0.003[-0.006:0.013]**	**0.71**	**0.480**
Specificity [95% CI]		**100.0 [69.2–100.0]**	**100.0 [69.2–100.0]**			
Cut off value**		**≥ 6.2**	**≥ 6.2**			
AUC	AUC [95%CI]	**1** [1]	**0.997 [0.857-1]**			
	SE	**0.00**	**0.006**			

*For the difference in AUC, ** at or above which event (i.e., disease) occurs

**Table 5:** ROC curve analysis (potentially malignant vs. malignant)

Parameter		Value	*z*-value	*p*-value*
Sensitivity [95%CI]		73.3% (44.9%:92.2%)	**5.41**	< 0.001*
Specificity[95%CI]		100.0% (76.8%:100.0%)		
Cut off value**		**≥** 7.9		
AUC	AUC [95%CI]	0.881 (0.707:0.971)		
	SE	0.07		

* For the significance of AUC, ** at or above which event (i.e., disease) occurs

## Discussion

The most prevalent form of head and neck tumors, OSCC, has drawn a lot of attention from scientific circles. This is a result of the disease’s extremely high incidence and mortality rates in many various nations across the world. That is, as well as the complicated social and economic effects it has on patients who are fortunate enough to survive this severely disabling condition [11].

As a result, early detection and treatment of oral cancer are critical for its successful management. A patient’s chance of survival may also increase with an earlier tumor diagnosis, which may help lessen therapy-related impairments in their ability to ingest food, swallow, and speak [23].

Although a biopsy remains the gold standard for diagnosing oral cancer, it is unsuitable for widespread screening purposes due to its invasive nature, high cost, and demand for adequately educated medical personnel and equipment [24]. Saliva, a fluid biological substance that is dynamic and comprised of various secretions, could potentially be viewed as a further non-invasive option [25]. Salivary biomarkers are considered a promising diagnostic supplement because they are used to screen a broad population and have a straightforward, non-invasive collection method [26].

Paxillin is a significant oncogenic factor in various malignancies, influencing cell communication, motility, growth, survival, vascular formation, and apoptosis [13, 19, 27]. Paxillin is a significant oncogenic factor in various malignancies, influencing cell communication, motility, growth, survival, vascular formation, and apoptosis [1]. Furthermore, it was discovered that paxillin levels are strongly linked to the development and dissemination of tumors. In comparison to patients who expressed low levels of paxillin, those who expressed high levels of paxillin had a diminished prognosis and life expectancy. Therefore, paxillin might be a promising therapeutic target for preventing the formation of tumors [19, 28–30].

The results of the present study reveal a statistically significant variation in salivary Paxillin concentrations among the three experimental groups (OPMDs, OSCC, and control). The OSCC group had the highest mean value, followed by the OPMDs group, whose healthy controls had the lowest salivary Paxillin levels. Additionally, results of the ROC curve analyses of the malignant group compared to the controls showed the highest sensitivity, and the area under the curve value was found to be 100%, which means that salivary paxillin can be used as an effective non-invasive tool to differentiate between the suspected oral malignant lesion and the normal control. Moreover, it has also been recorded that salivary paxillin has a high sensitivity to differentiate the OPMDs from the control, with the AUC value showing 93.3%. Both ROC curves showed cut-off values ≥ 6.2. Remarkably, results of ROC curve analysis between the OPMDs group and the malignant group elaborated the discriminative ability of Paxillin to differentiate well between both of them with a sensitivity of 73.3% and a cut-off value ≥ 7.9 .

Consistent with our findings, the study conducted by Aghili et al. [31] recorded the highest Paxillin expression in OSCC and dysplasia.They also determined that paxillin might be involved in the etiology of OSCC and the progression of dysplastic tissues. However, their study differs from the current one as they have investigated the paxillin levels by tissue sampling and not via salivary samples.

On the same line, Andisheh-Tadbir et al. [32] conducted a study to assess the tissue levels of paxillin in several salivary gland cancers, and they discovered that its greatest levels were detected in tumoral tissues compared to control, but its expression was not connected with the patients’ clinicopathological features.

Furthermore, equivalent results were shown by Song et al. [33] investigating the paxillin and FAK expression in cell culture and documenting an enhanced expression of paxillin levels in the OSCC cell line. Besides, they also concluded that the advanced expression of paxillin, FAK, and MMP-11 could strongly increase the incidence of oral cancer metastasis.

Remarkably, Shekhar and Angadi [12] evaluated and compared the paxillin levels in paraffin-embedded tissues of OSCC with different states of differentiation (well, moderate, and poor differentiation) in which paxillin-stain positivity was detected in 95.5% of the cases. Additionally, their research demonstrated that paxillin may contribute to the progression and development of OSCC.

Based on the findings of this investigation and prior similar studies, it could be concluded that Paxillin is a reliable biomarker that can be used to early detect and discriminate between OPMDs and malignant oral lesions via a non-invasive, simple salivary sample in which early diagnosis can be considered a crucial step for better prognosis as paxillin has also been involved in the progression, invasion, and metastasis of OSCC.

The limitation of the present research is represented in the small sample size, so an increased number of patients is recommended in future studies.

## Conclusions

In conclusion, the study offers compelling evidence supporting salivary paxillin as a reliable biomarker for oral cancer, emphasizing its potential clinical significance in early diagnosis and prognosis while advocating for further research to validate its broader diagnostic utility.

## Data Availability

The data supporting this study findings are available upon request from Dr. Yasmine Kamal yasmine.kamal@dentistry.cu.edu.eg as a corresponding author.

## Abbreviations

AUC: Area under the curve
ECM: Extracellular matrix
HRP: Horseradish Peroxidase
OD: Optical density
OL: Oral leukoplakia
OPMLs: Oral Premalignant and Malignant Lesions
OSCC: Oral squamous cell carcinoma
ROC: Receiver operating curve
SD: Standard deviation
